# Lineage-specific *tprK* diversification and *Treponema pallidum* transmission dynamics in Buenos Aires, Argentina

**DOI:** 10.64898/2026.01.29.702707

**Published:** 2026-01-30

**Authors:** Nicole A.P. Lieberman, Luciana N. Garcia, Shah A. K. Mohamed Bakhash, Jeff Furlong, B. Ethan Nunley, Andrés Rabinovich, Patricia Fernandez Pardal, Viviana Leiro, Hong Xie, Farhang Aghakhanian, Mauro Romero Leal Passos, Wilma Nancy Campos Arze, Hugo Boechat Andrade, Silver K. Vargas, Kelika A. Konda, Michael Reyes Diaz, Carlos Caceres, Jeffrey D. Klausner, Jonathan B. Parr, Arlene Seña, Ariyaratne Manathunge, Lorenzo Giacani, Jaime Altcheh, Alexander L. Greninger

**Affiliations:** 1 Department of Laboratory Medicine and Pathology, University of Washington School of Medicine, Seattle, WA, USA; 2 Servicio Parasitología-Chagas, Hospital de Niños Ricardo Gutierrez, Capital Federal, Buenos Aires, Argentina; 3 Instituto Multidisciplinario de Investigaciones en Patologías Pediátricas (IMIPP), CONICET, Buenos Aires, Argentina; 4 Hospital de Infecciosas F J Muñiz, Servicio de Dermatología, Buenos Aires City, Argentina; 5 Institute for Global Health and Infectious Diseases, University of North Carolina at Chapel Hill, Chapel Hill, NC, USA; 6 Department of Microbiology and Parasitology, Biomedical Institute, Fluminense Federal University, Niterói, RJ, Brazil; 7 Center for Interdisciplinary Studies in Sexuality, AIDS and Society, Universidad Peruana Cayetano Heredia, Lima, Peru; 8 Division of Infectious Disease, David Geffen School of Medicine, University of California Los Angeles, Los Angeles, CA, USA; 9 Keck School of Medicine, University of Southern California, Los Angeles, CA, USA; 10 Department of Medicine, Division of Infectious Diseases, Institute for Global Health and Infectious Diseases, University of North Carolina at Chapel Hill, Chapel Hill, NC, USA; 11 National STD/AIDS Control Programme, Ministry of Health, Colombo, Sri Lanka; 12 Department of Medicine, Division of Allergy and Infectious Diseases, Harborview Medical Center, University of Washington, Seattle, WA, USA; 13 Department of Global Health, Harborview Medical Center, University of Washington, Seattle, WA, USA; 14 Vaccine and Infectious Disease Division, Fred Hutchinson Cancer Center, Seattle, WA, USA

## Abstract

**Background:**

Syphilis rates are rising globally, with increases in congenital syphilis in South America particularly concerning. The characterization of contemporary South American *Treponema pallidum (Tp)* strains is crucial to syphilis vaccine development, yet few genomic epidemiology studies have focused on this region. Here, we performed whole genome sequencing of *Tp* from Buenos Aires, Argentina, as well as deep sequencing of the hypervariable *tprK* locus, which is critical to *Tp* immune evasion.

**Methods:**

People with primary, secondary, or congenital syphilis were enrolled at two clinics in Buenos Aires between October 2018 and January 2023, including individuals associated with intra-household transmission. *Tp* DNA from swabs was quantified by *tp0574* qPCR, and whole-genome sequencing was performed on samples with sufficient treponemal burden. *Tp* reads were assembled to the SS14 strain reference genome, recombinant regions masked, and a core genome phylogeny was generated. Full-length *tprK* was sequenced using PacBio reads.

**Findings:**

*Tp* genomes were recovered from 96 samples from 70 individuals in Buenos Aires and primarily belonged to globally dominant SS14 sublineage-1 and Nichols sublineage-8, as did *Tp* recovered from contemporary samples from Brazil (n=8). Peruvian samples (n=3) all belonged to sublineage-1. Two individuals from Buenos Aires had co-infections with Nichols- and SS14-lineage strains. Macrolide resistance via A2058G mutation occurred in 27/70 (39%) samples. Across 56 samples, *tprK* allelic diversity was significantly increased in secondary syphilis, oral lesions, and SS14-lineage strains compared to primary syphilis, anogenital lesions, and Nichols-lineage strains, respectively. Increased *tprK* diversity in SS14-lineage strains is driven by an enhanced repertoire of V7-specific donor sequences. Using multiple approaches, *tprK* sequences from intra-household transmission events were more similar than unrelated samples with identical core genomes.

**Interpretation:**

*Tp* circulating in South America is closely related to dominant global sublineages. Increased *tprK* diversity in the SS14 lineage may influence *Tp’s* ability to escape host adaptive immunity. We confirmed that *tprK* profiling is a promising tool to elucidate syphilis transmission networks. This study underscores the utility of genomics to yield insights into *Tp* pathogenesis.

## Introduction

In 2022, the Region of the Americas accounted for 42% (3.37 million) of all new syphilis diagnoses worldwide, representing the highest incidence of any World Health Organization region. Low- and middle-income countries in Central and South America account for most cases. The rate of syphilis in pregnant women in the Region increased 28% between 2020 and 2022, with 68,000 cases of congenital syphilis annually^1^ and in Argentina in 2023, 6% of women attending antenatal care tested positive for syphilis^2^. Until a vaccine that can prevent vertical transmission of *Treponema pallidum* subspecies *pallidum (Tp)* is available, reducing rates of congenital syphilis depends on identifying and breaking transmission chains, a process complicated by overlapping sexual networks and high rates of syphilis in vulnerable and hard-to-reach populations^3,4^. Despite the key role genomic epidemiology can play in understanding the spread and evolution of *Tp*, South American strains have been the subject of limited study.

Genomics-based studies of clinical samples can also shed light on the basic biology of *Tp*, which is difficult to study in the laboratory due to technically challenging cultivation and limited genetic tools^5,6^. *Tp* is notable for its minimalist 1.14 Mb genome and slow accumulation of genetic diversity, with fewer than 150 single nucleotide variants (SNVs) in the core genome separating strains of the Nichols and SS14 lineages of *Tp*^7,8^, which are clinically indistinguishable^9^. In contrast, ongoing gene conversion in *tprK* results in antigenic variation of seven extracellular loops of the *Tp* outer membrane protein TprK. Donor cassettes (DCs) from 53 chromosomal sites are recombined into the seven variable (V) regions to generate novel *tprK* alleles, tens or hundreds of which may be found in primary or secondary syphilis lesions^10,11^. Antigenic variation in *tprK* is ongoing during in vitro culture^12^ and can theoretically generate 10^13^-10^15^ unique mosaic alleles^10^ that can evade host adaptive immune responses^13,14^. Understanding *tprK* evolution can provide insight into *Tp*-immune dynamics that cause the relapsing clinical course of syphilis^14^. Additionally, *tprK’*s rapid evolution may be used to identify syphilis transmission networks^15^ where whole genome sequencing (WGS)-based methods lack resolution due to low diversity of the *Tp* core genome.

Here, we use WGS to genomically characterize contemporary *Tp* circulating in Argentina, Brazil, and Peru. We couple WGS analyses with investigations of *tprK* sequence diversity in the Buenos Aires cohort, which includes samples associated with previously-identified cases of intra-household syphilis transmission^16^, enabling us to extend our prior work using *tprK* to elucidate syphilis transmission networks^15^ as well as examine the effect of *Tp* genetic background on antigenic variation.

## Methods

### Ethics Approvals

In Buenos Aires, enrollment of pediatric participants and their caregivers (DI-2018–245-HGNRG) as well of adult participants (DI-2022–155-HGNRG) received approval from by the Committee and Review Board of the Hospital de Niños Ricardo Gutierrez. Sequencing of *Tp* from deidentified specimens was approved by the University of Washington IRB (STUDY00000885) and the University of North Carolina at Chapel Hill IRB (24–2696). Written consent was obtained from all adult participants and caregivers of enrolled children.

### Enrollment Criteria and Screening

A convenience sampling method was used to recruit patients in two public health services in Buenos Aires, Argentina between October 2018 and January 2023. A total of 94 adult outpatients were assisted in the Dermatology Unit of the Hospital Javier Muñiz, whereas another cohort of 47 patients, including pediatric outpatients (n=28) and their caregivers (n=19) were assisted at the Hospital de Niños Ricardo Gutierrez and studied as previously described^16^. Briefly, serological, epidemiological, and clinical data, including prenatal screenings and treatment data, were collected from the children and their cohabitants. A medical and psychosocial evaluation of the children and their caregivers was performed to evaluate sexual abuse^16^. Pediatric cases where sexual abuse was suspected were excluded. Additional screening criteria is found in Supplementary Methods, as are enrollment criteria for Brazil and Peru cohorts, which were included in WGS analyses.

### *T. pallidum* whole genome sequencing

WGS was performed as previously described^17^ on samples with more than 25 *tp0574* copies/μL DNA eluate, equivalent to a minimum of approximately 200 genome copies into library preparation. Briefly, genomic DNA was fragmented using the KAPA HyperPlus kit (Roche) and TruSeq unique dual indices (Illumina) ligated prior to 14–16 cycles of pre-capture PCR. Hybrid capture using our custom DNA probes was performed using the manufacturer’s instructions (Integrated DNA Technologies) prior to 14–16 cycles of post-capture amplification. Paired-end 150nt reads were generated on Illumina instruments including the HiSeqX, Novaseq6000, and NextSeq2000.

### *T. pallidum* genome assembly and phylogenetics

A total of 96 samples from 70 participants from Argentina, 8 samples from 8 participants from Brazil and 3 samples from 3 participants from Peru were newly sequenced for this study and included in a phylogenetic analysis alongside 1,772 publicly available genome sequences from the NCBI Sequence Read Archive (Supplementary Data). Genomes were assembled using a custom pipeline available at https://github.com/greninger-lab/tpallidum-variant-calling, outlined in Supplementary Methods.

### *tprK* sequencing and data pre-processing

The *tprK* gene was amplified from *T. pallidum* genomic DNA using 35 cycles of PCR with barcoded primers as previously described^17^ in technical duplicate and sequencing performed on the PacBio Revio. Following demultiplexing, HiFi reads were filtered to remove sequences shorter than 1400 bp or longer than 1700 bp or with unexpected primer orientations. Only reads with each base Q30 or higher were included in subsequent analyses. Variable regions and intervening constant regions were translated and reads containing frameshifts or premature stop codons in any region excluded from analysis. Custom analyses are described in Supplementary Methods.

### Statistics and Reproducibility

All calculations and statistics were performed in R v4.2.2. Custom processing scripts are available at https://github.com/greninger-lab/tprk-argentina/.

### Data Availability

All WGS data generated during this study are available at NCBI BioProject PRJNA723099, with accessions in Supplementary Data. *tprK* PacBio raw data are available in NCBI BioProject PRJNA1415869.

## Results

### Cohort demographics

A total of 141 patients (113 adults and 28 children) were enrolled, including seven individuals associated with three intra-household transmissions. Demographic characteristics of the adult and pediatric cohorts are summarized in Table 1. All adults were cisgender, with median age 26 years (IQR 22–33). All 45 men who have sex with men (MSM) were adult males, as were all 27 persons living with HIV (PLWH), including 5 individuals newly diagnosed during enrollment. Twenty-eight patients had prior syphilis. The pediatric cohort included four newborns with congenital syphilis, 12 children (5 males and 7 females) with a median age of 2 (IQR: 1–6) years and 12 adolescents (7 males and 5 females) with a median age of 16 (IQR: 15–17) years. Adolescents with primary and secondary syphilis were sexually active. Children with secondary syphilis have a median age of 3 years (IQRs: 2–6), caused by non-sexual close contact with active lesions from their caregivers^16,18^. Clinical findings are included in Supplementary Analysis.

### Yield of molecular testing and genome sequencing

A total of 250 samples were collected from the 141 enrolled patients, primarily oral and anogenital swabs (135/250, 54.0% and 92/250, 36.8% of samples, respectively). Molecular positivity for the single copy *tp0574 Tp* locus was highest among anogenital swabs (81/92, 88.0%), extragenital rash (9/13, 69.2%), and oral swabs (81/135, 60%). All 22 primary chancre swabs were positive. Two of thirteen (15.4%) blind swabs of normal mucosa in primary syphilis were *tp0547* positive. Among secondary syphilis, swabs of oral lesions were positive in 37 of 42 cases (88.1%), while blind swabs of normal oral mucosa were positive in 36 of 66 cases (54.5%) (Figure S1A). Primary lesion swabs had the highest interpolated *tp0574* copies per positive swab (median: 48,121 copies, n=16), followed by secondary disseminated anogenital lesions (median: 17,025 copies n=39) (Figure S1B). Specimens from patients that had had one or more prior episodes of syphilis (n=44 specimens from 28 patients) were less frequently *tp0574* positive than those who had no prior syphilis episodes (23/44, 52.3% vs 153/204, 75.0%), p=0.0047, chi-square test).

We attempted WGS on samples for which we could input at least 200 genome copies to library preparation. A total of 96 samples from 70 patients had sufficient coverage for analysis, which we defined as having less than 25% ambiguous bases in SNV positions. Among all sample types, the highest yield of near-complete genomes was from primary anogenital lesions (16/22, 72.7%; Figure S1C), followed by oral and anogenital lesions of the mucosa. No near-complete genomes were recovered from DNA extracted from whole blood. The proportion of genomes recovered among demographic groups was representative of the enrolled cohort (Table 1, all *p* >0.05, chi-square test), although no genomes were recovered from infants with congenital syphilis.

### Strains from Nichols and SS14 lineages of *T. pallidum* circulate in Buenos Aires

**Figure 1:**
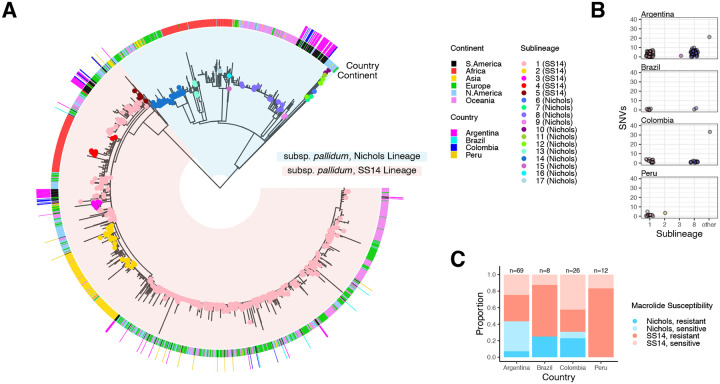
South American *T. pallidum* is closely related to globally dominant sublineages. A) Recombination-masked, maximum likelihood phylogeny of 1,879 *T. pallidum* strains, including 107 newly sequenced for this study. Blue background represents the Nichols lineage and red represents the SS14 lineage. Strains included in a study by Beale *et al* (2021) have tips colored by sublineage. The inner ring denotes continent of collection and outer ring denotes country for South American samples only. B) Number of single nucleotide differences between each South American sample and its most closely related worldwide sample, plotted by sublineage of the worldwide sample. C) Presence of A2058G mutation conferring macrolide susceptibility, by country and lineage.

Prior studies have shown that both SS14- and Nichols-lineage strains of multiple sequence types circulate in Buenos Aires^16,19^, however, South America is the least sampled of all continents other than Antarctica, and only a single South American sample was included in a recent comprehensive subclassification scheme^8^. Both lineages of *Tp*, which are named after the Nichols and SS14 strains, were detected in contemporary Colombian samples^9,20^. We built upon these studies by analyzing the genomes recovered from Argentine samples alongside publicly available South American *Tp* sequence data as well as three new samples from Peru and eight from Brazil. First, we contextualized South American strains within a worldwide phylogeny including a total of 1,879 *Tp* samples (Figure 1A). Most South American samples are closely related (within 10 SNVs) to previously classified samples in globally distributed sublineages 1 (SS14 lineage) and 8 (Nichols lineage)^8^ (Figure 1B). In Argentina, 32/71 (45.1%) individuals had Nichols-lineage treponemes, similar to Colombia (8/23, 34.5%). Nichols-lineage *Tp* has not been identified in Peru. Although genotypic macrolide resistance, conferred by A2058G mutation in the 23S rRNA gene, was relatively low in Argentina compared to other South American countries (39.1%; Figure 1C), these data reflect a progressive increase in resistance within Buenos Aires over the past decade^16,19,21^.

### No association between sublineage and demographic characteristics in Buenos Aires

**Figure 2:**
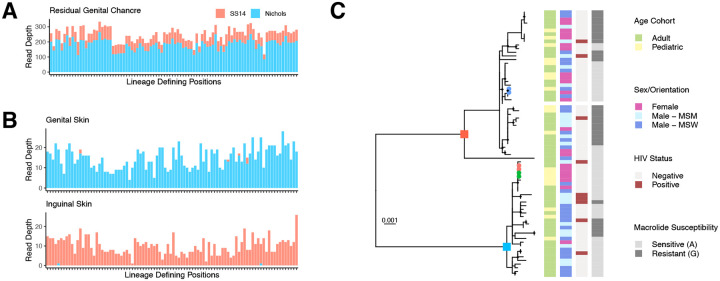
Co-infection and *Treponema pallidum* genomic epidemiology within Buenos Aires, Argentina, 2018–2023. A) Whole genome sequencing read depth shows the presence of ~80% Nichols- and ~20% SS14- variants at lineage-defining positions, supporting a co-infection in the residual genital chancre of an individual (GSPAR041) with secondary syphilis. B) Secondary syphilis lesions from genital and inguinal skin of another individual (GSPAR083) belong to Nichols and SS14 clades, respectively. Individuals in both A and B had a history of multiple sex partners. C) Phylogeny of strains collected in Buenos Aires, 2018–2023. Nichols and SS14 lineage ancestral nodes are labeled with blue and red squares, respectively. Tips from samples of each patient within the three household transmission clusters are colored red, green, and blue, respectively. Tiles correspond to patient age, sex and sexual orientation, HIV status, and macrolide susceptibility (A2058G).

Although co-infection with both Nichols- and SS14-lineage strains has only been reported once before^22^, two individuals in the Buenos Aires cohort had evidence for co-infection. A sample from a residual genital chancre in an individual with secondary syphilis who reported multiple sex partners in the preceding three months yielded mixed SS14- and Nichols-like sequence reads at lineage defining positions^8^ (Figure 2A). In another person, also reporting multiple sex partners, SS14- and Nichols-lineage strains were recovered from different lesions (Figure 2B). Co-infections were confirmed by strain-specific digital PCR^22^ using DNA re-extracted from the original swab. In one of 20 individuals with WGS from multiple secondary syphilis lesions, we found compartmental SNVs in *tp0006*, a gene of unknown function (Figure S2, Supplementary Analysis).

We constructed a phylogeny of *Tp* genomes from Buenos Aires, including strains from our current study and one sample collected in 2016^15^ (n=73 genomes from 71 individuals, Figure 2C). Thirty-one samples formed nine distinct clusters with 0 core-genome SNVs, including clusters of individuals within households as well as clusters with no known epidemiologic link. Among individuals without Nichols-SS14 co-infection and with clinical information (n=68), we did not find any association between patient sex, sexual orientation, or HIV status with *Tp l*ineage. Macrolide resistance was more prevalent in the SS14 lineage than the Nichols (22/38, 57.9% vs 5/30, 16.7%, p<0.01), and in MSM (11/17, 64.7%) compared to MSW or females (10/29, 34.5% and 6/22, 27.3%, respectively, p<0.05). Mutations to 16S rRNA sites homologous to those conferring tetracycline resistance were not observed^23,24^.

### The number and distribution of *tprK* alleles depends on stage, site, and *T. pallidum* lineage

We next interrogated the hypervariable *tprK* gene using high fidelity PacBio sequencing coupled with our customized analyses (Figure S3). TprK undergoes antigenic variation within seven of its extracellular loops to evade host adaptive immunity^13,14,25–27^ by replacing fragments of *tprK* with donor cassettes (DCs) from 53 chromosomal regions arranged in two loci (Figure S4). Variable regions were defined essentially as described previously^10,25^, however, for ease of interpretation we split variable and constant regions between codons. Sequences between variable loops exhibited minimal variation, with no changes to a known B cell epitope^28^ (Figure S5, Supplementary Analysis).

**Figure 3:**
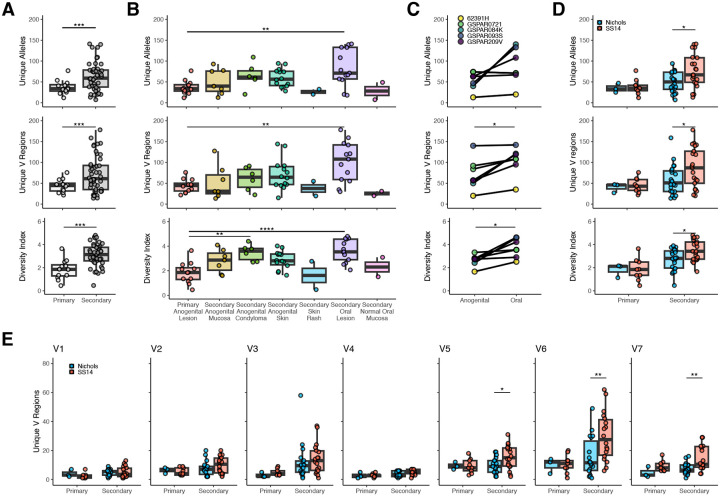
Number and distribution of *tprK* alleles. A) Differences in total alleles (top), unique variable regions (middle), and Shannon diversity index (bottom) by syphilis stage. T-test, ***p<0.001. B) Differences in *tprK* by lesion type and location. ANOVA with Tukey’s post hoc, **p<0.01, ****p<0.0001. C) Samples from individuals with paired samples from anogenital and oral sites show a higher number of V regions and diversity index in the oral lesion. Paired T-test, *p<0.05. D) *tprK* diversity in Nichols vs SS14 lineage samples, t-test, *p<0.05. E) Variation by clinical stage and lineage in the number of unique V regions. T-test, *p<0.05, **p<0.01.

We examined the relationship between clinical and demographic factors in the number of unique *tprK* alleles, V regions, and Shannon diversity index in each sample, including a total of 56 samples from 44 individuals with sufficient read depth and quality (Figure S3). V regions were only counted if present in both technical replicates. We did not limit inclusion of full-length *tprK* alleles to only those present in both technical replicates because it had a different effect on primary and secondary samples (Figure S6A–B), consistent with differences in the underlying distribution of alleles between primary and secondary syphilis^11^. No full-length *tprK* alleles recovered were shared between patients. Among ten individuals with *tprK* sequencing from more than one body site, six paired samples shared one or two full-length *tprK* alleles. The number of *tprK* alleles exceeding 0.2% abundance ranged between 7–141 per sample. Consistent with prior reports^10^, secondary syphilis samples of all types yielded a significantly higher number of unique full-length *tprK* alleles (median 63 vs 37, p <0.001, Figure 3A) as well as more V regions and higher Shannon diversity index. When lesion type and location was considered, oral secondary lesions had significantly higher *tprK* diversity than anogenital primary lesions, though all secondary lesion types except rash had a higher median number of alleles and greater diversity than primary lesions (Figure 3B). Among the five individuals from whom paired oral and anogenital lesions of any type were collected, oral lesions consistently had higher *tprK* diversity than anogenital lesions (Figure 3C). Among 55 samples from 43 patients with paired WGS and full-length *tprK* profiling, SS14-lineage samples had more unique *tprK* alleles than those from the Nichols lineage (Figure 3D), driven primarily by increased diversity in V6 and V7 (Figure 3E, Figure S7). Among samples with paired WGS, a total of 3,142 unique *tprK* alleles were found, including 1,191 in Nichols-lineage samples (n=25) and 1,951 in SS14-lineage samples (n=30). Patient sex, sexual orientation, prior syphilis, HIV coinfection, and age were not associated with differences in *tprK* diversity, though prior syphilis and PLHV were very small groups (Figure S8).

### SS14 lineage strains generate *tprK* diversity with an expanded repertoire of *tprK* donor cassettes

To further explore the observation of increased *tprK* diversity in the SS14 lineage, we applied a k-mer-based analytical approach designed to account for its extensive rearrangement. *tprK* evolves via recombinogenic changes that replace segments of tens of nucleotides in each variable region^25^ (Figure S3B), precluding the use of maximum-likelihood methods to determine sequence relatedness. Therefore, we examined differences in *tprK* sequence composition between lineages by first breaking each sequence into short overlapping subsequences (k-mers) of lengths ranging between 9–27nt (Figure S9), allowing comparison of sequence similarity despite extensive recombination and rearrangement. We focused particularly on k-mers of length 21 nt since the seven-amino acid fragments (7AA-mers) they encode approximate the minimal length of a linear B cell epitope^29^, yet are short enough to enable identification of *tprK* sequence fragments that that are exact matches to chromosomal donor cassettes^12,25^. Although some could theoretically have been generated via gene conversion to stitch donor sequences together, we will define *tprK* 21-mers that perfectly match one or more donor cassettes as “donor cassette (DC)-derived”. K-mers and AA-mers found only in *tprK* sequences from strains of the Nichols or SS14 lineage are defined as “lineage-specific”.

**Figure 4:**
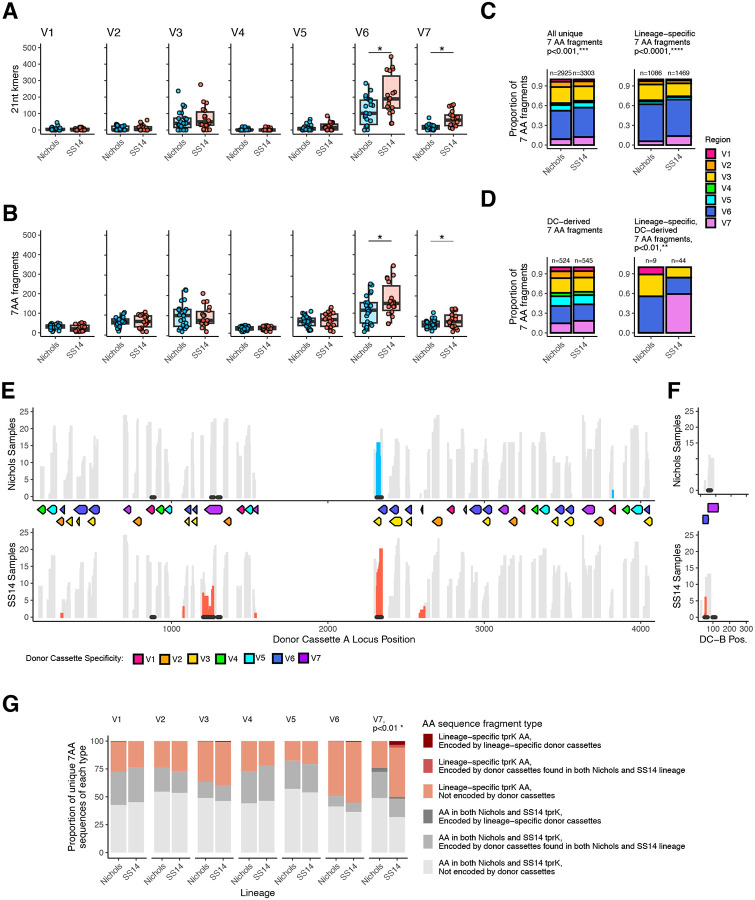
Lineage-specific differences in *tprK* sequence content. All analyses in this figure were performed by sampling an equal number of samples from each lineage (25) followed by random subsampling of 1,000 unique alleles per lineage. Significance is defined as a mean p<0.05 across 1,000 independent subsampled replicates. A single representative replicate is visualized. The total number of unique V6 and V7 21 nt k-mers (A) and 7AA-mers (B) are significantly higher in SS14 than Nichols lineage. T-test, *p<0.05. C) The proportion of unique 7AA-mers derived from each region differs by lineage among all 7AA-mers as well as those found in only the SS14 or Nichols lineage. ***p<0.001 and ****p<0.0001, chi squared. D) The proportion of 7AA-mers derived from each region and encoded directly by donor cassettes is not different between SS14 and Nichols lineages, but each lineage contains a different proportion of lineage-specific 7AA-mer fragments directly encoded by donor cassettes, **p<0.01, chi squared. SS14 uses more lineage-specific donor cassette (DC)-derived sequences than Nichols (44 vs 9). E) Histogram of the number of Nichols (top) or SS14 (bottom) samples containing at least one *tprK* allele using DC-derived 7AA-mer from each donor site in DC-A and DC-B. Locations of donor cassettes colored by V region specificity are shown. DC-derived AA-mers found in *tprK* sequences from both Nichols and SS14 lineages are shown in grey. Lineage-specific 7AA-mers are shown in blue (Nichols) and red (SS14). Locations of chromosomal sequence differences between Nichols and SS14 donor cassettes are marked with black points. G) Proportion of unique 7AA-mers of each type – present in both lineages, Nichols only, or SS14 only, and directly encoded by DCs or a mosaic sequence not found in the donor cassettes – by variable region. SS14 lineage strains contain significantly more lineage-specific V7 7AA-mers than Nichols, including both DC-derived and mosaic sequences generated by gene conversion, **p<0.01, chi squared.

To ensure balanced representation of 21-mer diversity, SS14 samples (n=30) were first down-sampled to the same number (25) as Nichols, then 1,000 *tprK* alleles from each lineage randomly selected from the 25 samples. SS14 lineage samples had more V6 and V7 21-mers and 7AA-mers than Nichols (Figure 4A–B), with a considerably greater proportion of unique SS14 7AA sequences derived from V7 than in Nichols (Figure 4C). While SS14 and Nichols strains had similar proportions of 7AA-mers encoded by 21-mers that were exact matches to those encoded by donor cassettes (DCs), SS14 strains had significantly more lineage-specific DC-derived 7AA fragments than Nichols (44 vs 9, p<0.01), half of which were found in V7 (Figure 4D).

We subsequently determined whether the increased diversity in the SS14 lineage strains could be accounted for by differences in the loci that contribute to recombination. We previously showed how differences in donor repertoire can affect *tprK* diversity both by genetically knocking out the major DC locus^27^ and detecting a clinical strain missing nine sets of DCs that had a reduced number of *tprK* variable regions relative to wild type strains^15^. Among strains in this analysis, the predominant DC alleles were lineage-specific, with unique mutations between SS14 and Nichols lineages. Differences between Nichols and SS14 loci were confined to SNVs in donor cassettes specific for V3, V6, and V7, as well as SNVs between DCs (Figure S10). When the DC-derived, lineage-specific 21-mers were mapped to their respective donor cassette locus, a majority of strains harbored at least one *tprK* allele with a lineage-specific 7AA-mer derived from the V3/V6 overlapping donor cassettes 26 and 25 (Figure 4E). The largest difference between Nichols and SS14 donor cassette alleles is a 51bp indel in V7-DC31 (Figure S10), which directly contributes novel sequences to *tprK* alleles in SS14-lineage samples. When assessing both the type (DC-derived or not) and lineage specificity of unique *tprK* k-mers, we found that SS14 has significantly greater use of lineage-specific V7 7AA fragments than Nichols (Figure 4G). Interestingly, the lineage-specific DC sequences found in Nichols V7-DC31 and V7-DC2 are not found in Nichols *tprK* alleles, while they are readily found in SS14 (Figure 4E–F), thus accounting for the reduced diversity of the V7 *tprK* allele seen in Nichols strains. Together, these data suggest that the increased *tprK* sequence diversity observed in SS14-lineage strains is due in part to an enhanced repertoire of V7 donor cassettes.

### *tprK* is more similar between individuals within known transmission chains than between unrelated individuals

**Figure 5:**
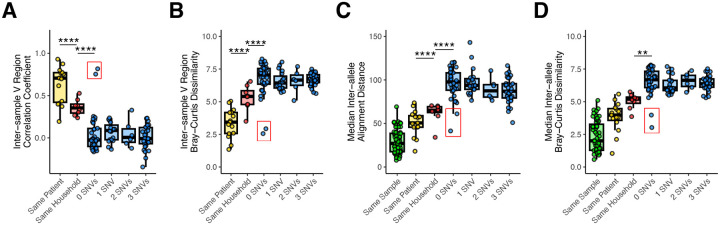
Inter-sample *tprK* relatedness by pairwise sample relationship. Pairwise comparisons of *tprK* profiling data were performed using different statistical methods were performed. Samples were grouped based on whether they were taken from different samples from the same individual, individuals from the same household constituting likely transmission, and based on core genome SNV distance. A) Pearson coefficient of relative proportions of variable regions. B) Composite Bray-Curtis Dissimilarity for DNA kmers in variable regions. C) Median pairwise alignment distance between *tprK* alleles in each sample pair type. D) Median composite inter-allele composite Bray-Curtis dissimilarity in each sample pair type. ANOVA with Tukey’s post hoc, **p<0.01, ****p<0.0001. Red boxes enclose samples from a pair of patients that may be epidemiologically linked based on patient age, sex and orientation, and presentation to the clinic on the same day,

Finally, we extended our previous findings showing that *tprK* sequences shared by two individuals are more similar in patients with a suspected epidemiologic link^15^, enabling outbreak tracing. First, we recapitulated our prior results showing that the Pearson correlation between the proportion of V regions between samples is greater within known intra-household transmission events than in epidemiologically unrelated samples with identical core genomes (Figure 5A, p<0.05, t-test)^15^. Next, we increased the specificity of the comparison by determining the inter-sample Bray-Curtis dissimilarity for each V region and length of k (Supplementary Methods). Each variable region had a differential ability to discriminate between samples from the same patient, same household, or unrelated individuals (Figure S11). V1 and V4 have lower Bray-Curtis dissimilarity between samples from the same household than unrelated individuals, while V6 only has low k-mer dissimilarity in samples from the same individual. These results are consistent with prior observations that V1 and V4 evolve the most slowly and V6 evolves the most rapidly, resulting in the highest diversity among V regions^12^. We calculated a composite Bray-Curtis dissimilarity score that included k-mers from all seven V regions, summed across all values of *k* to account for both the high sensitivity of short kmers and high specificity of longer kmers (Figure 5B). We also calculated the median inter-allele global alignment distance between *tprK* alleles within and between each sample (Figure 5C) as well as the median inter-allele composite Bray-Curtis score (Figure 5D). All methods showed a significant difference in *tprK* similarity between known transmission chains (intra-household) versus epidemiologically unrelated samples. Analysis of full-length alleles (Figure 5C–D) did not further resolve distinctions between known transmission events and unrelated samples relative to methods relying only on total V region content (Figure 5A–B).

Interestingly, among samples separated by 0 SNVs, all methods identified unexpectedly low Bray-Curtis dissimilarity between the oral and genital samples from one patient and the genital sample from a second patient (Figure 5, red boxes). Notably, outside of the V regions, strains from both patients also had a W516C mutation in the C-terminus of TprK (Figure S5B). Upon further review of patient clinical data, we found that the samples came from a male with secondary syphilis and a female with primary syphilis. Both patients were in their mid-20s, heterosexual, denied stable sexual partners, and visited the clinic on the same day. Together, these findings support the utility of *tprK* sequencing in identifying syphilis transmission chains for epidemiological investigation.

## Discussion

As the syphilis epidemic in South America continues to accelerate, sequencing of *Tp* provides considerable insight into syphilis epidemiology, revealing that most strains isolated in South America are closely related to those circulating throughout the world^8^. We also extend our previous methods using *tprK* similarity to elucidate transmission events, which can augment public health responses by detecting network links not captured by traditional contact tracing approaches^3^. Using multiple independent methods to profile *tprK* relatedness in a unique cohort of samples that included household linkage, we show *tprK* sequences from individuals within a transmission chain are more closely related than those from unrelated samples with identical core genomes. Moreover, the method revealed a possible transmission event that had not been recorded clinically, showing the power of this technique to identify closely related cases that merit follow-up public health investigation. Analysis of full-length *tprK* alleles did not demonstrate a significantly enhanced ability to resolve inter-sample distances relative to analysis of V regions alone, suggesting that *tprK* profiling could be deployed using short-read sequencing technologies that have been widely adopted in public health labs. The sensitivity of *tprK* profiling to unmask possible transmission links has important implications for patient privacy, a consideration for future study design and use by public health departments.

An important limitation of the current study of *tprK* is the use of convenience sampling from a single location, Buenos Aires, resulting in a relatively small number of participants for a very diverse metric like *tprK*. In particular, the number of known transmission events (three) was very low. Furthermore, the household transmission samples – which are suspected to be acquired syphilis by nonsexual contact^16,18^ - may not be fully representative of *tprK* evolution during sexual transmission. Nonetheless, the work described here replicates and significantly extends prior *tprK* profiling in Seattle, Washington^15^.

In addition to the key public health questions that can be answered using *Tp* WGS and targeted *tprK* sequencing, genomic studies of *Tp* evolution in patient samples play an important role in revealing aspects of pathogenesis and evolution that are challenging to study in vitro given limited experimental tractability and technically challenging cultivation. Unexpectedly, we showed that *tprK* recovered from oral lesions is more diverse than from anogenital lesions, even from patient-matched specimens, which may reflect different immune surveillance or other selection pressures at different mucosal sites. Future investigations of subtle differences in *tprK* diversity will require incorporating unique molecular identifiers or other techniques to better resolve rare alleles, approaches we are currently developing. Additionally, we demonstrate for the first time increased *tprK* diversity in the SS14 lineage relative to the Nichols lineage and attribute it to an increased repertoire of unique donor sequences resulting from a 51 bp insertion in a V7-specific donor cassette. As this increased diversity in the SS14 lineage results in multiple novel potentially antigenic variants not seen in the Nichols lineage, enhanced *tprK* diversity may provide a fitness advantage to SS14 strains by enabling more variants to escape adaptive immune responses to *tprK*, potentially accounting for SS14’s enhanced prevalence globally.

In conclusion, we find WGS coupled with *tprK* profiling to provide extensive insight into *Tp* pathogenesis. Furthermore, the use of inter-sample Bray’s dissimilarity scores of *tprK* V regions is a promising tool to infer syphilis transmission networks. Future work involving a wider range of sampling, including more known transmission events, will be necessary to confirm these findings and further refine methods to infer transmission events from *tprK* profiling. Critically, studies that connect *tprK* variation with host immune responses will be essential to define how antigenic variation shapes immune evasion by SS14 and Nichols lineage *Tp*.

## Supplementary Material

1

## Figures and Tables

**Table 1:** Demographic characteristics by cohort.

	Adult patients (n=113) n (%)	Pediatric patients (n=28) n (%)	Total Patients (n=141) n (%)	Patients with WGS (n=70) n (%)	p value*
**AGE: median (IQRs) years**	26 (22–33)	6 (2–16)	24 (19–31)	27 (18–33.5)	
Adults (>18)	113 (100.0)	-	113 (80.1)	51 (72.8)	0.31
Pediatric (0–18)	-	28 (100.0)	28 (19.9)	19 (27.2)	
**BIOLOGICAL SEX**					
Male	78 (69.0)	13 (46.4)	91 (64.5)	48 (68.6)	0.67
Female	35 (31.0)	15 (53.6)	50 (35.5)	22 (31.4)	
**SEXUAL ORIENTATION (among adult and adolescent males)**					
MSM	45 (57.7)	0 (0)	45 (52.9)	19 (43.2)	0.39
No MSM	33 (42.3)	7 (100%)	40 (47.1)	25 (56.8)	
**HIV**					
Positive	26 (23.0)	0 (0)	26 (18.4)	9 (13.8)	0.41
Negative	87 (77.0)	28 (100%)	115 (81.6)	61 (86.2)	
**PRIOR SYPHILIS**					
Yes	27 (23.9)	1 (3.6)	28 (19.8)	10 (14.2)	0.28
No	85 (75.2)	27 (96.4)	112 (79.4)	70 (85.8)	
Not specified	1 (0.9)	0 (0)	1 (0.7)	0 (0)	
**CLINICAL STAGE**					
Primary	21 (18.6)	3 (10.7)	24 (17.0)	16 (22.8)	0.24
Secondary	92 (81.4	21 (75.0)	113 (80.1)	54 (77.2)	
Congenital syphilis	0 (0)	4 (14.3)	4 (2.8)	0 (0)	

* The p value was derived from a chi-square test comparing the proportion of patients with sequenced genomes to the total patient population.

## Research in context

### Evidence before this study:

Although whole genome sequencing (WGS) and genomic epidemiology have contributed to an understanding of the global diversity of *Treponema pallidum* (*Tp*), very few strains from South America have been sequenced to date. On January 6^th^, 2026, we performed a PubMed search including terms “syphilis genomic epidemiology”, “South America”, “*Treponema pallidum*”, and “Argentina”. We excluded studies of ancient *Tp* samples found in South American archeological sites. A single *Tp* sample from Argentina was originally sequenced in 2016 and included in subsequent analyses of global diversity. Nine Peruvian samples were previously sequenced in a study of global diversity on six continents. Thirty-three samples from Cali, Colombia were predominantly SS14-lineage and genotypic macrolide resistance was found in half of strains. Additionally, studies of *Tp* in Buenos Aires using a multi-locus sequence typing approach have shown circulation of strains belonging to both Nichols and SS14 lineages, with an increasing rate of macrolide resistance over time. Aside from a report of inter-strain recombination of *Tp* in an individual infected with both Nichols- and SS14-lineage strains, no studies have found intra-host evolution of *Tp* outside of the *T. pallidum* repeat (tpr) family or acidic repeat proteins. Five previous studies have examined *tprK* in clinical specimens and consistently shown increased *tprK* diversity in specimens associated with secondary syphilis compared to primary syphilis lesions. One prior study has shown that loss of *tprK* donor cassettes is associated with reduced *tprK* diversity, consistent with results from in vitro experiments. A single study has shown that *tprK* sequence content is more similar in samples with a suspected epidemiologic link, though no prior studies have looked at *tprK* diversity in the context of known syphilis transmission events.

### Added value of this study:

This study is the first to use WGS to comprehensively examine *Tp* transmission in a large South American city, yielding 96 samples from 70 individuals. We also add the first contemporary *Tp* genomes from Brazil. In contrast to findings from England, Australia, and other high-income countries, no *Tp* sublineages are associated with particular demographic groups or sexual networks in Buenos Aires. Two of seventy patients were co-infected with both Nichols- and SS14-lineage strains, showing the previously unappreciated frequency of conditions that permit inter-strain recombination-driven diversification of *Tp*. This study also reveals novel aspects of *Tp* pathogenesis, including recovery of specific intra-host *tp0006* point mutations from different specimen types and lineage-specific differences in *tprK* diversity. We also develop methods for the analysis of *tprK* relatedness between samples and demonstrate that *tprK* sequences are more similar in samples from individuals within intra-household syphilis transmission chains compared to those from epidemiologically unrelated individuals.

### Implications of all the available evidence:

*Tp* strains circulating in Buenos Aires,are genetically similar to those circulating worldwide and in Brazil and Peru but are noteworthy for the low (but rising) rate of macrolide resistance. Lineage-specific patterns of *tprK* antigenic variation could result in differences between Nichols- and SS14-lineage strains’ interactions with the host immune system. Finally, we show that *tprK* profiling holds promise to identify samples from within a syphilis transmission chain and could play an important role in public health.
